# Host restriction factors against porcine epidemic diarrhea virus: a mini-review

**DOI:** 10.1186/s13567-025-01500-4

**Published:** 2025-03-24

**Authors:** Hongqing Zheng, Cunyi Qiu, Haolun Tian, Xiaofu Zhu, Baoying Yin, Zhiding Zhou, Xuezhao Li, Jingjing Zhao

**Affiliations:** 1 Xianyang Polytechnic Institute, Xianyang, 712000 China; 2 Gansu Polytechnic College of Animal Husbandry & Engineering, Wuwei, 733006 China; 3 Northwest a&F University, Yangling, 712000 China; 4 Key Laboratory of Marine Animal Disease Prevention and Control, Dalian Ocean University, Dalian, 116023 China; 5 Department of Pharmaceutical Engineering, School of Biomedical and Pharmaceutical Sciences, Guangdong University of Technology, Guangzhou, 510006 China

**Keywords:** PEDV, swine, host restriction factor, virus–host interaction

## Abstract

Porcine epidemic diarrhea is an acute contagious disease caused by porcine epidemic diarrhea virus (PEDV), which severely constrains the development of the global swine industry. Host restriction factors constitute a vital defensive barrier against viral infections, typically interacting with viruses at specific stages of their replication process to disrupt it. Considering that traditional PEDV vaccines often struggle to effectively activate mucosal immunity in sows and thereby fail to provide reliable passive immunity to piglets via milk, this review focuses on the host restriction factors that play crucial roles in restricting PEDV infection and replication. The aim is to identify potential targets for the development of anti-PEDV drugs and offer insights for the exploration of novel vaccine adjuvants.

## Introduction

During prolonged interactions between the body and viruses, host cells produce a variety of antiviral molecules known as host restriction factors. These proteins, found within the host cells, inhibit viral replication and combat viral infection [1]. They function as cellular defense proteins of the innate immune system, typically targeting multiple pathogens and playing a crucial antiviral role at various stages, including virus adsorption, invasion, replication, assembly, and release [2, 3] (Table 1).

**Table 1:** **Host restriction factors—summarized according to the life cycle of the virus**

The virus life cycle	Target protein	Action mechanism	Related host restriction factors
Adsorption	–	Inhibition of virus adsorption	MUC2
	S		DNAJA3
	APN		ADAM17
Viral entry	CLTC	Inhibition of PEDV endocytosis	Mortalin
Virus replication	TRAF6	Inhibition of virus replication	KLF16
	TRAF3		ATG4B
	PERK, IRE1		GRAMD4
	STAT3		PTPN14
	–		DAXX, p53
	RdRP	Inhibition of viral genome replication	CMPK2
Virus maturation and release	ORF3	Inhibit virus assembly and release	VPS36
	E	Inhibit virus assembly and budding	KPNA2, eIF3L
	N	Inhibit the assembly of virus particles	IRAV, hnRNP K, FUBP3, TARDBP, BST2, PRPF19, PTBP1, PABPC4, RALY, RBM14, TRIM21

Viruses are non-cellular life forms composed of a nucleic acid chain and a protein shell (capsid). Lacking metabolic machinery and enzymatic systems, viruses rely entirely on host cells to reproduce [4]. The innate immune response serves as the primary defense against viral invasion. Upon viral entry, pattern recognition receptors detect viral pathogen-associated molecular patterns, triggering an antiviral innate immune response [5]. Given the structural characteristics of viruses and their interaction with the host immune system, restriction factors exert antiviral functions in several ways. For instance, the restriction factors IRAV [6] and hnRNP K [7] degrade certain crucial viral proteins through the ubiquitin–proteasome and autophagy–lysosome pathways. These proteins are typically intimately associated with viral replication or assembly [8]. Apoptosis can contribute to the release of viral particles from infected cells; however, premature apoptosis and cellular disintegration can limit the time and space available for virus replication [9]. Host restriction factors such as GRAMD 4 [10] specifically target the apoptosis pathway to induce apoptosis in infected cells, effectively clearing the virus and playing an important role in defense against viral infections. The PEDV is a single-stranded positive-sense RNA virus whose genome replication relies on an RNA-dependent RNA polymerase. Host restriction factors such as CMPK2 [11] inhibit viral genome replication by suppressing RNA-dependent RNA polymerase activity. Further, certain host restriction factors can activate innate immune pathways and produce interferons (IFNs) [12], which stimulate the expression of IFN-stimulated genes via paracrine or autocrine pathways to enhance antiviral effects. For example, PTBP1 [13] and TARDBP [14] boost host innate immune responses by upregulating MyD88 expression.

Porcine epidemic diarrhea virus (PEDV), a member of the *Alphacoronavirus* genus within the *Coronaviridae* family [15]. The PEDV S protein is recognized as the primary inducer of neutralizing antibodies in the host; however, it is also among the most highly glycosylated proteins in nature, with numerous glycosylation sites composed of high mannose. During viral evolution, such sites are susceptible to insertions, deletions, and mutations, which can diminish the cross-immunoprotective capabilities of vaccines, posing a significant challenge in the development of effective PEDV vaccines [16]. In recent years, researchers have identified various host restriction factors that effectively inhibit the replication and infection of PEDV. However, the antiviral mechanisms of only a few of these factors have been extensively studied. Investigating and characterizing the host restriction factors involved in PEDV infection and the mechanisms by which they inhibit viral replication can enhance our understanding of PEDV–host interactions.

This article provides a comprehensive review of the research progress on host restriction factors that affect the infection and replication cycle of PEDV. The findings of our study offer potential targets for the development of anti-PEDV drugs and provide new insights into the research and development of novel PEDV vaccine adjuvants.

## Host restriction factors impair PEDV replication by targeting the innate immune pathway

**Figure 1:**
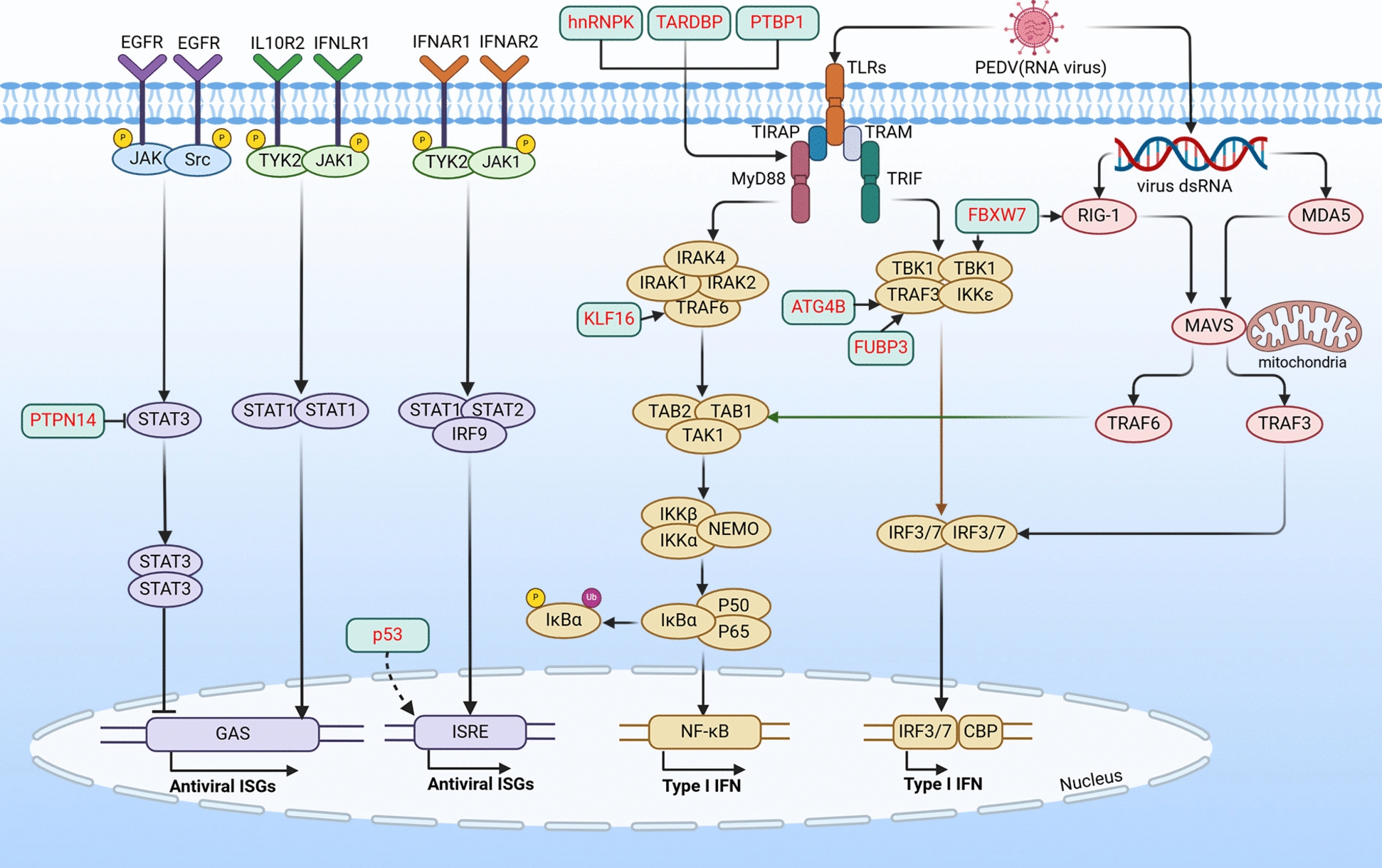
**Host restriction factors impair PEDV replication by targeting the innate immune pathway.** “⊣” Denotes the inhibition or degradation of downstream molecules. “→”Indicates the activation of downstream molecules. “X” Indicates removal of the original effect. Red text represents the host restriction factor.

Innate immunity serves as the host's first line of defense against viral infections. Following viral infection, host immune cells promptly produce IFN-I, which triggers the expression of various IFN-stimulated genes, enabling the body to enter an antiviral state [17, 18] (Figure 1). The toll-like receptors (TLRs) constitute the earliest-discovered family of innate immune receptors and recognize a broad spectrum of pathogen-associated molecular patterns (PAMPs) [19]. Previous studies have implicated TLR2, TLR3, TLR4, TLR7, and TLR9 in NF-κB activation in porcine intestinal epithelial cells (IECs) induced by PEDV, suggesting that PEDV employs its envelope glycoproteins and nucleic acids on its surface to trigger innate immunity [20, 21].

TLR signaling pathways can be classified into MyD88-dependent and MyD88-independent pathways (TRIF-dependent pathways). All members of the TLR family, except TLR3, initiate signal transduction by recruiting the MyD88 protein to the C-terminus of the TIR domain [22]. MyD88 encompasses a death domain and recruits interleukin-1 receptor-associated kinases (IRAKs) to form the Myddosome signaling complex. In the Myddosome, IRAK4 is activated through trans-autophosphorylation and subsequently phosphorylates IRAK1 [23]. Activated IRAK1 disengages from the Myddosome and complexes with TRAF6, resulting in TRAF6 dimerization and triggering its E3 ubiquitin ligase activity.

The E3 ubiquitin ligase activity of TRAF6 is crucial for TAK1 activation. The host restriction factor FBXW7 is also an E3 ubiquitin ligase that promotes the ubiquitination and subsequent degradation of multiple proteins, enhances RIG-I and TBK1 expression, induces interferon gene expression, and serves as a positive innate antiviral effector [24]. The primary role of TAK1 is to activate the classical IκB kinase (IKK) complex and initiate NF-κB nuclear translocation and interferon gene transcription [25]. The host restriction factors TARDBP and PTBP1 activate the IFN-I signaling pathway by up-regulating MyD88 expression [13, 14], while KLF16 up-regulates TRAF6 expression in a dose-dependent manner, ultimately leading to IFN activation and thereby restricting PEDV replication [8].

TLR3 participates in the innate immune response via a TRIF-dependent pathway. Specifically, TRIF interacts with TRAF3, which recruits the IKK-related kinases TBK1 and IKKε and activates IRF3 through serine/threonine phosphorylation. IRF3 then forms a dimer and translocates from the cytoplasm to the nucleus to induce IFN-I gene expression [22]. The host restriction factor ATG4B activates IFN-I signaling by up-regulating TRAF3 expression, thereby restricting PEDV replication [26].

Death domain-associated protein (DAXX), a classical chaperonin involved in apoptosis, transcriptional regulation, DNA damage repair, and host innate immunity, inhibits PEDV replication. DAXX activates the IFN-λ3-STAT1 signaling pathway and upregulates ISG15 expression [27]. STAT3, a downstream effector molecule of various cytokines and growth factors, typically inhibits IFN-I antiviral activity and facilitates viral infection [28]. Li et al. [29] found that STAT3 tyrosine phosphorylation is critical for PEDV infection in host cells. To counteract viral infections, the host employs a complex mechanism to inhibit STAT3 activation. Specifically, PTPN14, a non-receptor tyrosine phosphatase significantly upregulated in PEDV-infected Vero cells, impedes STAT3 phosphorylation and activation, thereby enhancing the IFN-I pathway [30]. p53, a tumor suppressor gene, can be activated under various conditions, such as DNA damage or stress. p53 overexpression activates IFN signaling and IFN-stimulated response elements, thereby inhibiting PEDV replication [31].

However, PEDV can also evade innate immunity by hijacking certain host proteins. For instance, PEDV upregulates TRIM28 expression in host cells to subsequently facilitate its own replication [32]. Mechanistically, the TRIM28 protein interacts with PEDV-N through its RING domain, enhancing mitophagy and inhibiting the JAK/STAT1 signaling pathway, leading to the downregulation of interferon-stimulated genes (ISGs) such as ISG15 and MX1. Zinc-finger antiviral protein (ZAP), a component of pattern recognition receptors, recognizes CpG-rich viral RNAs and exerts antiviral effects by initiating RNA degradation processes. TRIM25 mediates the K63-linked polyubiquitination of ZAP, essential for the RNA-binding capability of ZAP. PEDV-N competitively binds the SPRY domain of TRIM25, preventing TRIM25–ZAP interactions and inhibiting the antiviral activity of ZAP [33].

## Host restriction factors impair PEDV replication by targeting the autophagy–lysosomal pathway

**Figure 2:**
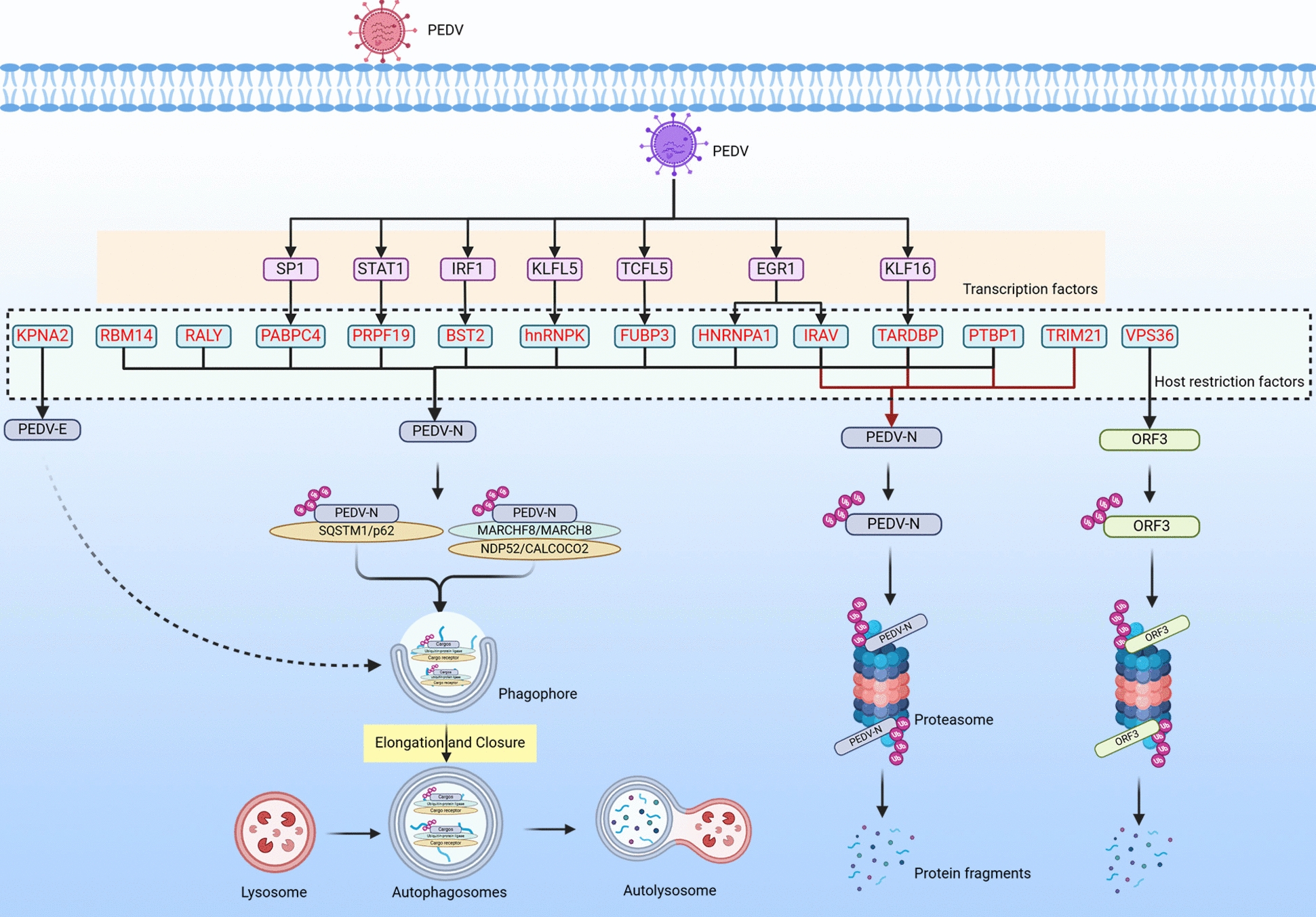
**Host restriction factors impair PEDV replication by targeting autophagy–lysosomal and ubiquitin–proteasome pathways.** “⊣” Denotes the inhibition or degradation of downstream molecules. “→” Indicates the activation of downstream molecules. “X” Indicates removal of the original effect. Red text represents the host restriction factors.

Autophagy was initially regarded as a non-selective degradation process triggered by cellular starvation. However, continued research has recognized that autophagy targets specific substrates through a series of specific cargo receptors in selective autophagy processes [34]. These processes typically involve the following steps: (i) cargo receptors receive signals indicating the decomposition of their substrates and then bind to molecular chaperones (such as E3 ubiquitin ligases); (ii) autophagy cargo molecular chaperones promote post-translational modifications of the cargo, making it recognizable by multiple cargo receptors; and (iii) cargo receptors transport the substrates to autophagosomes for degradation. Depending on the substrates being degraded, selective autophagy can be classified as xenophagy, mitophagy, aggrephagy, reticulophagy, lipophagy, and ferritinophagy, among others. [34]. Xenophagy, as an innate immune response at the cellular level, plays a role in restricting pathogen proliferation by targeting and degrading certain key protein components of invading pathogens [35] (Figure 2).

The PEDV N protein can bind and package viral genomic RNA into nucleocapsids, playing a crucial role in the assembly process of virus particles [36]. Selective autophagy receptors (SARs), also known as cargo receptors, play a vital role in the process of selective autophagy degradation, responsible for connecting autophagy substrates and autophagosomes. p62 was the first discovered autophagy cargo receptor [34], and host restriction factor RNA-binding motif protein 14 (RBM14) degrades the PEDV N protein by recruiting p62 [37]. The NDP52 autophagy cargo receptor also plays an important role in xenophagy of invading pathogens. Its C-terminus contains a UBZ domain that can bind to ubiquitin proteins, specifically recognizing ubiquitinated invading pathogens (proteins), thereby mediating their selective autophagy and clearance [38]. The host restriction factors heterogeneous nuclear ribonucleoprotein K (hnRNP K) [7], heterogeneous nuclear ribonucleoprotein A1 (HNRNPA1) [39], far upstream element-binding protein 3 (FUBP3) [14], cytoplasmic poly(A) binding protein 4 (PABPC4) [40], HnRNP-associated with lethal yellow (RALY) [41], pre-mRNA processing factor 19 (PRPF19) [42], and bone marrow stromal cell antigen 2 (BST2) [43] can ubiquitinate the N protein by recruiting the E3 ubiquitin ligase MARCH8. Subsequently, the ubiquitinated N protein is transported to autophagosomes by the cargo receptor NDP52 for selective degradation, thereby inhibiting PEDV replication. However, all seven host restriction factors mentioned above other than BST2 are RNA-binding or RNA-processing proteins. With respect to these types of proteins, we typically focus more on their roles in enhancing mRNA stability and promoting protein translation. Whether they play a key role in restricting PEDV replication and whether they have the potential to become targets for antagonizing PEDV replication still requires further investigation.

The envelope protein is the smallest structural protein in PEDV and is involved in processes such as viral particle assembly and budding [44]. Recent studies have found that the host restriction factor karyopherin α2 (KPNA2) inhibits PEDV replication by targeting E protein degradation through autophagy. However, the specific ubiquitination process and the recruited cargo receptors need further investigation [45].

## Host restriction factors impair PEDV replication by targeting the ubiquitin–proteasome pathway

In eukaryotic cells, the ubiquitin–proteasome system (UPS) serves as the primary pathway for the modification and degradation of cellular proteins, playing a pivotal role in regulating cellular physiological activities. Additionally, it is closely associated with the lifecycle of viruses and their interaction with the host [46] (Figure 2). The nuclear localization of the N protein can activate the p53–DREAM signaling pathway, leading to S-phase arrest in host cells and creating a favorable cellular microenvironment for PEDV replication; therefore, the N protein plays a significant role in PEDV replication [47]. Host restriction factors, such as IFN-regulated antiviral (IRAV) [6] and tripartite motif-containing 21 (TRIM21) [48], can interact with the PEDV N protein by recruiting the E3 ubiquitin ligase MARCH8, resulting in N protein degradation via the ubiquitin–proteasome pathway and ultimately inhibiting PEDV replication.

ORF3, the only accessory protein encoded by the PEDV genome, forms a protein channel structure. Although direct evidence linking ORF3 ion channel activity to PEDV replication is currently lacking, ORF3 may indirectly promote PEDV replication by regulating the cell cycle to prolong the S-phase [49], inhibiting early apoptosis [50], and promoting autophagy [51]. Most coronaviruses enter host cells through the endocytic pathway [52]. Vacuolar protein sorting-associated protein 36 (VPS36) mediates protein degradation during the fusion of multivesicular bodies with lysosomes [53]. VPS36 degrades ORF3 through the proteasome pathway, thereby inhibiting PEDV replication [54].

## Host restriction factors impair PEDV replication by targeting the apoptosis pathway

**Figure 3:**
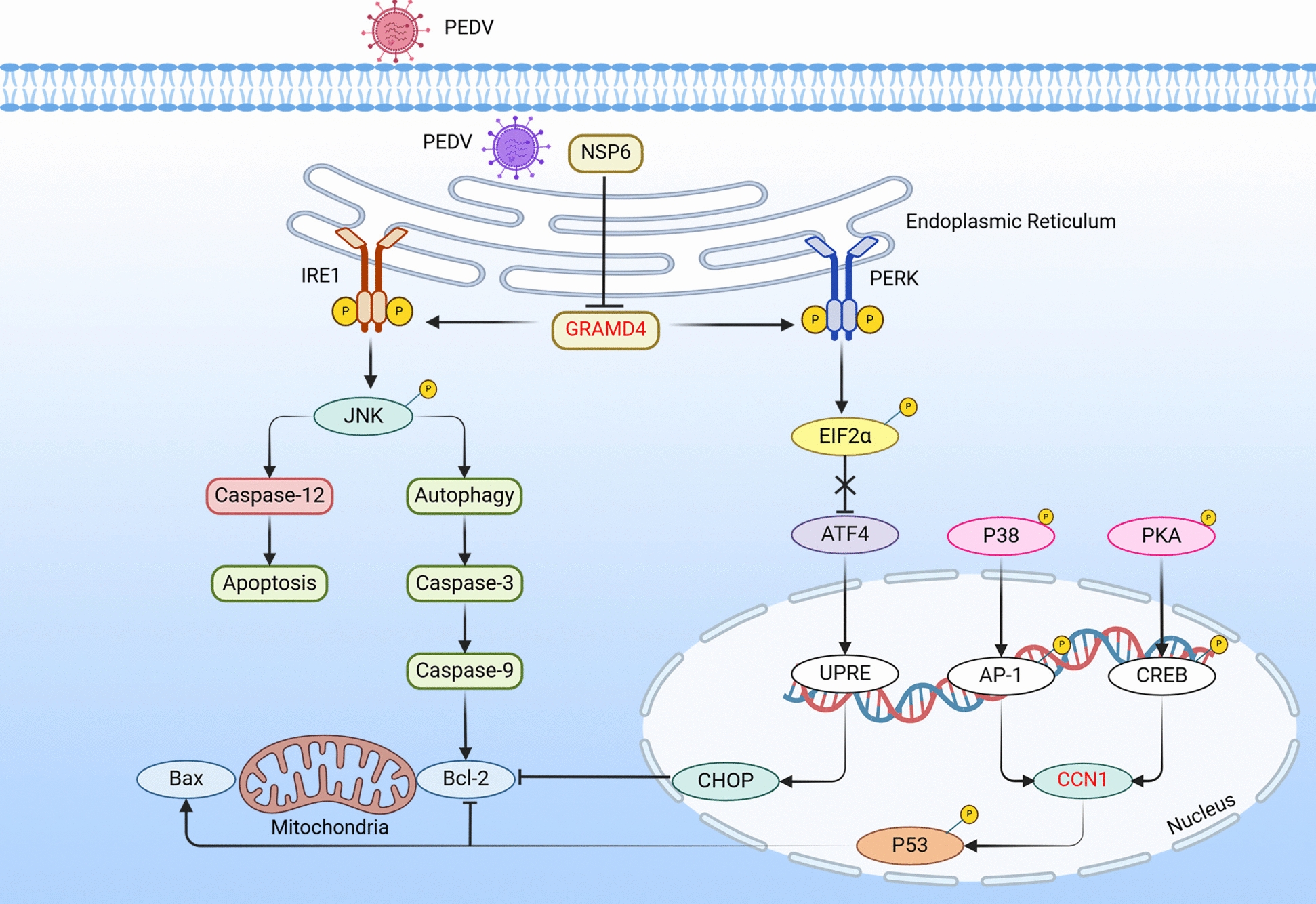
**Host restriction factors impair PEDV replication by targeting and modulating the apoptosis pathway**. “⊣” Denotes the inhibition or degradation of downstream molecules. “→” Indicates the activation of downstream molecules. “X” Indicates removal of the original effect. Red text represents the host restriction factor.

Apoptosis is a physiological defense mechanism that regulates viral proliferation. During viral infection, certain host restriction factors hinder viral replication within the body by enhancing cellular apoptosis [55]. Extracellular matrix proteins, which are apoptosis-related factors, play a role in the onset of apoptosis [56] (Figure 3). As an extracellular matrix protein, cellular communication network factor 1 (CCN1) promotes apoptosis by activating p53 and downregulating survivin expression in esophageal adenocarcinoma tumor cells [57]. Zhou et al. [58] demonstrated that PEDV activates the transcription factors CREB and AP-1 via the PKA and p38 pathways, respectively. These factors bind to the CCN1 promoter and upregulate CCN1, which regulates apoptosis by promoting p53 phosphorylation, thereby inhibiting PEDV replication. The nonstructural PEDV protein NSP6 downregulates the expression of glucosyltransferase Rab-like GTPase activator and myotubularin domain-containing 4 (GRAMD4), a pro-apoptotic protein [59], via the PERK and IRE1 pathways, and GRAMD4 overexpression effectively inhibits PEDV replication. Moreover, the GRAM domain is essential for GRAMD4 to promote ER stress-mediated apoptosis and restrict viral replication [10].

## Host restriction factors impair PEDV replication by targeting additional pathways

DnaJ heat shock protein family (Hsp40) member A3 (DNAJA3) interacts with the middle fragment (amino acids 378–479) of the PEDV S1 protein, affecting viral adsorption to IPEC-J2 cells and thereby inhibiting virus replication [60].

The MUC2 protein is the main component of intestinal mucus secretion and is primarily involved in cell signal transduction and immune regulation [61]. Pretreatment with 50 μg/mL porcine mucin 2 can reduce PEDV infection in host cells [62]. Biering et al. recently demonstrated that membrane-anchored mucins 1 and 4 can prevent SARS-CoV-2 from binding to cell surfaces [63]. However, when the cells were treated with esterase inhibitors that selectively cleave mucins, the number of viral particles bound to their surfaces increased significantly.

Tight junctions (TJs) are important junction complexes between epithelial cells and are composed of transmembrane (occludin and claudin) and perimembrane (ZO) protein families. TJs serve as paracellular barriers that maintain cellular polarity. Par-3 family cell polarity regulator (PARD3), a polarity-related protein closely related to TJs, belongs to the post-synaptic density protein-95/Discs large/Zonula occludens-1 protein family [64, 65]. PARD3 is primarily located in the periphery of the cytoplasm and cellular membrane and regulates epithelial cell polarity, particularly in maintaining apical–basal polarity [66]. Many viruses target basolateral receptors during infection; however, the TJ barrier renders these receptors inaccessible, preventing viruses from completing the infection process [67]. However, some viruses have developed mechanisms that disrupt TJ barriers. For example, during PEDV infection, PARD3 is degraded via a proteasome-dependent pathway. Furthermore, downregulating PARD3 disrupts the TJs of cells and affects the polarization of the tops and bottoms of the cells, thereby promoting PEDV infection [30].

Porcine aminopeptidase N (APN) is a key cell surface receptor for PEDV infection [68]. Disintegrin and metalloprotease 17, a well-characterized member of the ADAM family, is known for its role in mediating the cleavage of various cell surface proteins [69, 70]. Zhang et al. showed that ADAM17 overexpression suppresses APN expression on the cell surface, consequently limiting PEDV invasion and restricting PEDV replication [62]. Mortalin, also known as mtHsp70/PBP74/Grp75/HSPA9, is a member of the heat shock protein (Hsp) 70 family. Mortalin plays multiple roles in membrane-mediated macromolecule transport, endocytosis, and exocytosis. Mortalin induces CLTC degradation through the proteasome pathway, thereby inhibiting the clathrin-mediated endocytosis of PEDV into host cells [71].

**Figure 4:**
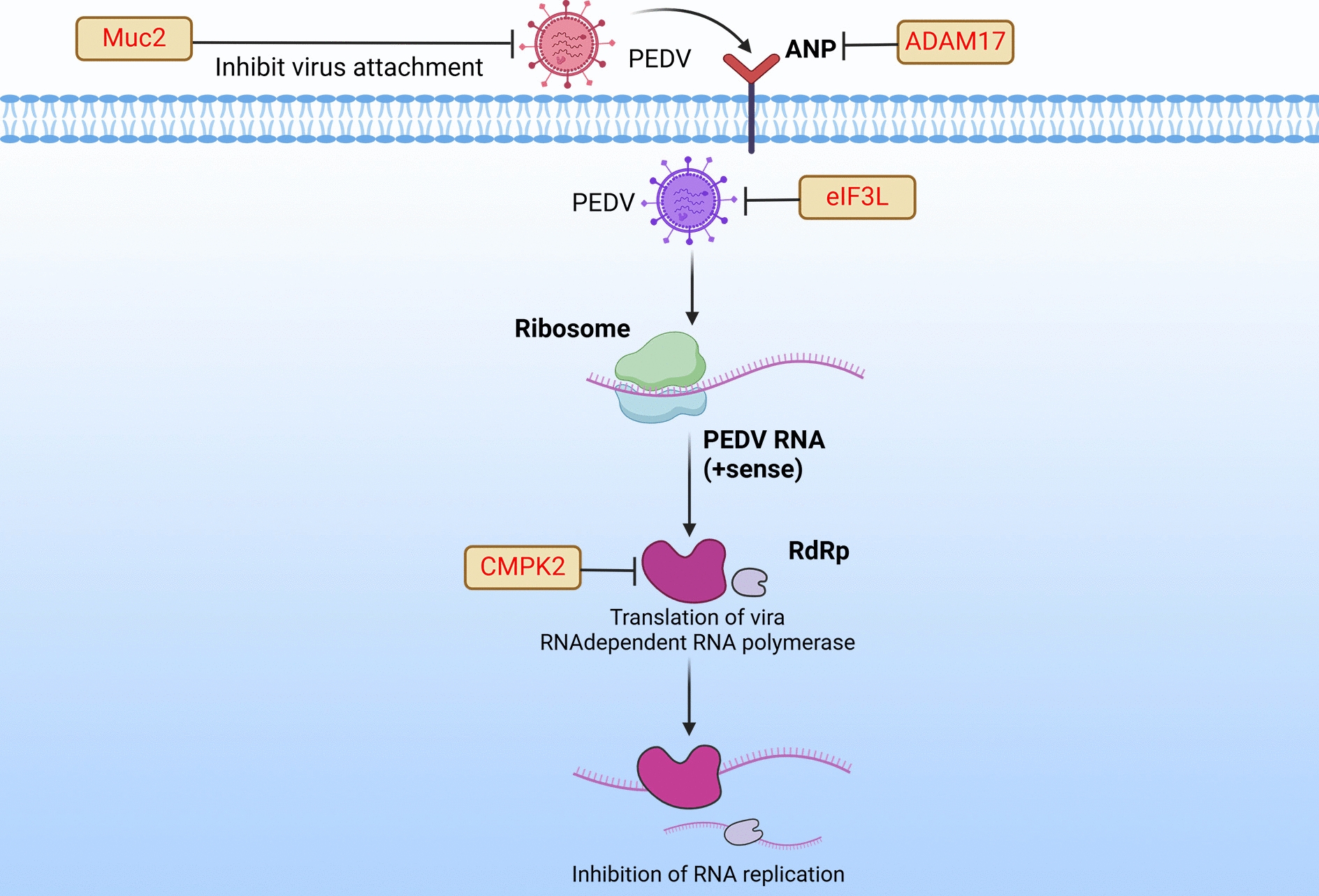
**Host restriction factors impair PEDV replication by targeting additional pathways.** “⊣” Denotes the inhibition or degradation of downstream molecules. “→” Indicates the activation of downstream molecules. “X” Indicates removal of the original effect. Red text represents the host restriction factor.

The coronavirus genome encodes 16 nonstructural proteins, among which, the RNA-dependent RNA polymerase encoded by nsp12 is pivotal in viral RNA replication and transcription. This enzyme is considered one of the most important antiviral drug targets [72]. Cytidine monophosphate kinase 2 (CMPK2), an interferon-stimulated gene (ISG) induced by IFN-I, inhibits the replication of various viruses, including human immunodeficiency virus, spring viremia of carp, and dengue virus [73–75]. As a potential host restriction factor, CMPK2 exerts anti-PEDV infection activity across various cell types. This activity is modulated by 3′-deoxy-3′,4′-didehydro-cytidine triphosphate (ddhCTP), produced by another interferon-stimulated protein, viperin. After PEDV infection, CMPK2, viperin, and ddhCTP act to inhibit PEDV replication by inhibiting RNA-dependent RNA polymerase activity [11] (Figure 4).

Coronaviruses such as herpesviruses [76], lentiviruses [77], bornaviruses [78], and filoviruses [79] represent an ancient lineage of viruses that have coexisted with their natural hosts, including birds and bats, for hundreds of millions of years [80]. Over this extended period of coexistence, hosts have evolved various factors to resist viral infections. Among the 24 host restriction factors reviewed in this article, 14 inhibit PEDV replication by targeting the autophagy–lysosome pathway or the ubiquitin–proteasome pathway. This suggests that these two pathways, as the two most important protein degradation pathways in eukaryotic cells, play crucial roles in providing inherent antiviral defense for the host [81–83].

As more host restriction factors are discovered, they offer potential targets for the development of anti-PEDV drugs. However, viruses have evolved a series of strategies to counteract the inhibitory effects of these host factors during their evolutionary arms race with their hosts. For instance, during PEDV infection, the host restriction factor Sin3-associated protein 18 (SAP18) promotes the dephosphorylation and activation of RIG-I, which is essential for the degradation of viral RNA mediated by the RIG-I-MAVS pathway. Nevertheless, PEDV Nsp10 reduces the expression level of SAP18 and induces its cytoplasmic accumulation [84]. Additionally, the PEDV E protein can induce the formation of stress granules by upregulating G3BP1 expression, leading to global inhibition of host cell protein synthesis during the translation phase [85]. Therefore, activating the expression of host restriction factors to maximize their antiviral effects has become a key focal point of our research.

On the contrary, delving into the mechanisms of host restriction factors will facilitate the development of vaccines and novel adjuvants. As an integral part of the host's inherent antiviral machinery, host restriction factors play a significant role in limiting viral replication [86]. However, this can be detrimental to increasing vaccine antigen content. Thus, in the vaccine production process, studying how to relieve the inhibitory effects of host restriction factors on virus proliferation is of practical importance for enhancing vaccine antigen content. Furthermore, host restriction factors play a crucial role in regulating immune responses [87]. The preparation of immune-related host restriction factors as vaccine adjuvants co-presented with vaccine antigens to stimulate a stronger immune response may also emerge as a significant research direction for host restriction factors.

## Data Availability

All data generated or analysed during this study are included in this published article.
